# Positron scattering from interstellar phosphorus-bearing compounds†

**DOI:** 10.1039/d4ra06809b

**Published:** 2024-12-10

**Authors:** Irabati Chakraborty, Nafees Uddin, Bobby Antony

**Affiliations:** a Department of Physics, Indian Institute of Technology (Indian School of Mines), Atomic and Molecular Physics Laboratory Dhanbad – 826004 Jharkhand India bobby@iitism.ac.in; b Applied Science Department, JIMS Engineering Management Technical Campus 48/4, Knowledge Park III Greater Noida 201308 Uttar Pradesh India

## Abstract

This article reports an investigation of positron impact scattering from several phosphorus-bearing compounds detected in the interstellar medium. The targets studied are HCP, CCP, CP, PN, and PO. For our theoretical model, we employed the spherical complex optical potential (SCOP) and complex scattering potential – ionization contribution (CSP-ic) methods. We computed the positron impact integral cross-sections for a wide range of energies, from 1 eV to 5000 eV in a fine energy grid. These cross-sections include positronium formation, direct ionization, total ionization, elastic, differential, and total cross-sections. We compared our newly reported data to molecules with similar structures to assess the quality of the calculated data. These results provide a valuable benchmark for future experimental and theoretical research on these targets.

## Abstract 2

This article reports an investigation of positron impact scattering from several phosphorus-bearing compounds detected in the interstellar medium.

## Graphical abstract

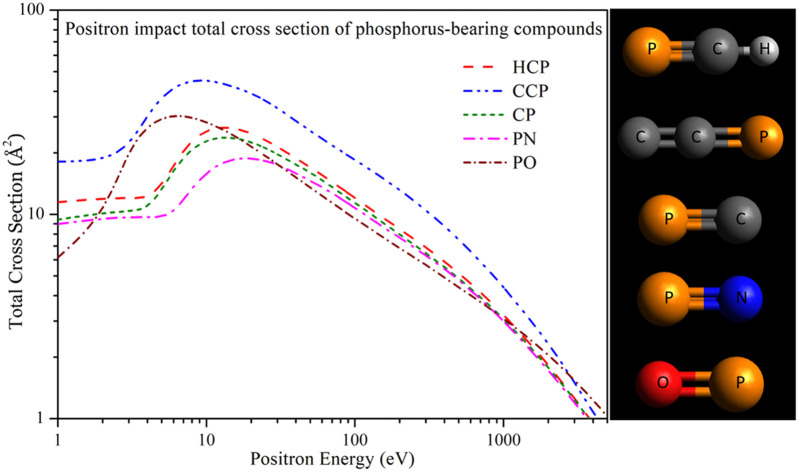

## 1 Introduction

Phosphorus is a critical element in the chemistry of life, serving as a foundational component of key biological molecules.^1^ It is a key component of DNA and RNA, the molecules responsible for storing and transmitting genetic information in all living organisms.^2^ Phosphorus is also a major part of ATP (adenosine triphosphate), the primary energy carrier in cells, facilitating energy transfer and storage necessary for numerous cellular functions.^3^ Furthermore, phospholipids contribute to the structure of cell membranes, ensuring cellular integrity and function. The prebiotic importance of phosphorus lies in its ability to facilitate energy transfer and biochemical reactions, making it indispensable for the emergence and sustainability of life.^4^ The versatility of phosphorus in forming various chemical compounds under prebiotic conditions has led to the hypothesis that it could have been a key element in the early stages of biochemical evolution.^5,6^ The widespread distribution of phosphorus on Earth reflects its fundamental role in biological and geological processes.^7^ Recent studies have identified several phosphorus-bearing compounds, such as PO (phosphorus monoxide), PN (phosphorus mononitride), CP (carbon monophosphide), CCP radical, and HCP (phosphaethyne), in the gas phase of circumstellar envelopes around evolved stars.^8^ Among these, PO and PN have been detected in star-forming regions.^9^ Therefore, the study of phosphorus in prebiotic chemistry is not only vital for understanding the origin of life on Earth but also for exploring the potential for life elsewhere in the universe.^10^

The first detection of a phosphorus-bearing compound in space began with PN (phosphorus mononitride), which Turner and Bally discovered in 1987 in the Orion Molecular Cloud using millimeter-wave observations.^11^ Since then, PN has been detected in several star-forming regions and remained the only phosphorus-containing species identified in dense interstellar mediums (ISM) for many years,^12–14^ until PO (phosphorus monoxide) was discovered by Rivilla *et al.* in 2016 in massive star-forming regions, along with PN, using the IRAM 30 m telescope.^9^ Guélin *et al.* subsequently identified CP (carbon monophosphide) in the circumstellar envelope of the star IRC+10 216 in 1990.^15^ Agúndez *et al.* discovered HCP (phosphaethyne) in the circumstellar envelope of IRC+10 216 using millimeter-wave spectroscopy.^16^ Halfen *et al.* detected the CCP radical in IRC+10 216 in 2008.^17^ These discoveries have significantly contributed to our understanding of phosphorus chemistry in space.

The study of positron scattering with molecules plays a pivotal role in bridging fundamental physics with practical applications across various fields. These studies enhance our understanding of fundamental interactions by resolving how positrons, the anti-particles of electrons, interact with matter. In recent decades, there has been increased interest in positron scattering from various targets, owing to its contributions across several fields, including medical sciences, atomic physics,^18,19^ astrophysics,^20,21^ and spectroscopy.^22,23^ Positron–molecule interaction cross sections are crucial for understanding particle track simulations.^24^ These cross sections are fundamental inputs for modeling software like GEANT,^25^ PENELOPE,^26^ EPOTRAN,^27^ and LEPTS,^28^ which use them to estimate radiation-induced damage. Positron Emission Tomography (PET)^29,30^ is a powerful medical imaging technique used to observe metabolic processes in the body. Oncology, neurology, and cardiology widely use it for diagnosing and monitoring diseases such as cancer, Alzheimer's disease, and heart conditions. In PET imaging, cross-section data is essential for modeling the scattering behavior of positrons, enabling more accurate simulations of their trajectories.^31,32^ In the past, positron scattering experiments were challenging to conduct, primarily due to limited access to high-resolution positron beams and the complexity involved in distinguishing the different partial cross sections. Thus, theoretical studies of positron scattering cross sections are essential for advancing science, interpreting astronomical data, improving material research, and guiding experimental efforts.

**Fig. 1:**
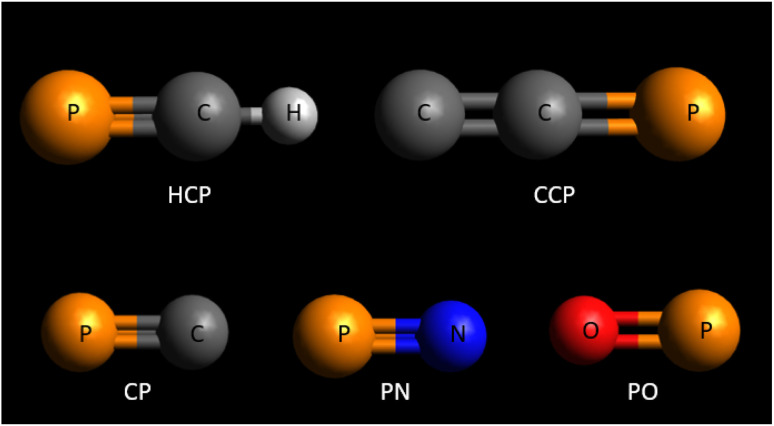
Structure of the phosphorus-bearing compounds studied in the present work.

In this work, we focused on the positron impact cross sections for a set of phosphorus-bearing compounds that have been identified in the interstellar medium. The specific compounds examined include HCP, CCP, CP, PN, and PO. The structure of these phosphorus-bearing compounds are given in Fig. 1. To achieve a comprehensive analysis, we utilized well-established theoretical models, the spherical complex optical potential (SCOP), and the CSP-ic (complex scattering potential – ionization contribution) methods. These two methods have been widely used to calculate electron^33–37^ and positron^38–40^ scattering cross sections for various atomic and molecular targets across a wide range of energy. Given that there are no previous experimental or theoretical studies available for these specific targets, we compared our findings with those of molecules that possess similar structures and bonding characteristics. This approach allowed us to validate our results against known data, even though direct comparisons for the exact compounds were not possible. The different types of positron impact cross sections that we computed in our study are total cross sections, positronium formation cross sections, direct ionization cross sections, elastic cross sections, total ionization cross sections, and elastic differential cross sections. We conducted these calculations across an extensive energy range, ranging from 1 eV to 5000 eV. This broad energy span ensures that our results cover a significant portion of the relevant physical processes involved in positron interactions with these phosphorus-bearing compounds. The present study aims to provide detailed data that will serve as a benchmark for future experimental and theoretical investigations in the field. By offering a comprehensive set of cross-section data, our findings will aid researchers in validating and refining their models and experiments, ultimately advancing the understanding of positron–molecule interactions in various scientific and practical applications.

## 2 Theoretical methodology

This study employs a modified version of the well-established optical potential formalism, SCOP, along with the CSP-ic method^38–40^ to compute various cross-sections. A single-center additivity rule (SCAR) is considered for all the molecules, as these targets are compact in structure. In the SCAR method, the molecular charge density and potential are obtained from the atomic charge densities and potentials by expanding them from the center of mass of the respective target before calculating the cross sections of the molecule. In the SCOP formalism, the positron–target interaction is modeled by a complex potential,

1 *V*_opt_ = *V*_R_ + i*V*_I_ = *V*_st_ + *V*_pol_ + i*V*_abs_

Which includes the static potential (*V*_st_), the polarization potential (*V*_pol_), and the absorption potential (*V*_abs_). The real part of the potential equation governs the elastic processes during a positron–molecule collision. The static potential originates from the coulomb interaction between the incoming positron and the target's unperturbed electron cloud. In this study, the static potential is determined using the parameterized Hartree–Fock (HF) wavefunctions provided by Cox and Bonham.^41^ Because of its charge, the incoming positron perturbs the target's charge cloud, resulting in the polarization potential. Zhang *et al.* introduced the parameter-free model of correlation polarization potential *V*_pco_(*r*),^42^ which describes both short-range correlation and long-range polarization effects, in conjunction with Perdew and Zunger's proposed correlation potential *V*_co_(*r*).^43^ The mathematical form of this correlation-polarization potential proposed by Zhang *et al.* is described as,2
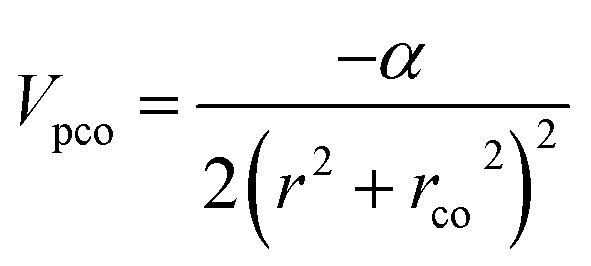
where *α* is the polarizability of the target. The value of *r*_co_ can be determined by putting 
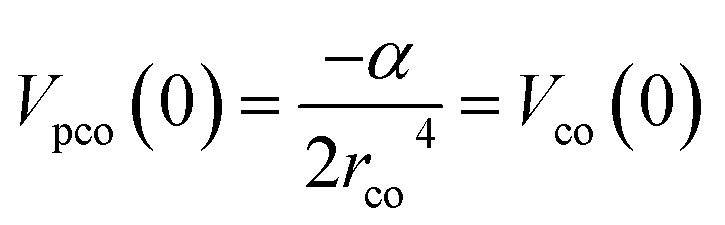
, which makes *V*_pco_(*r*) and *V*_co_(*r*) equal at the origin. In the near target region, *V*_pco_(*r*) approaches *V*_co_(*r*) and at large *r*, it takes the asymptotic form −*α*/2*r*^4^. The mathematical form of the correlation potential *V*_co_(*r*) proposed by Perdew and Zunger is given as,3
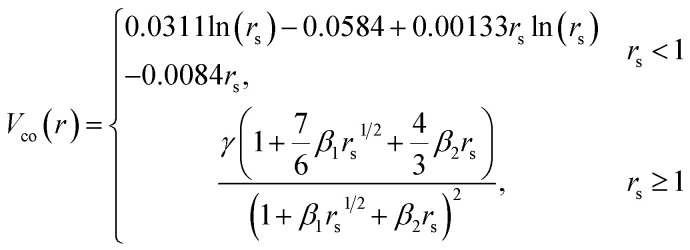
where 
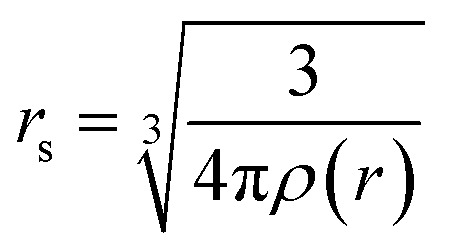
 is the density parameter, *ρ*(*r*) is the charge density of the target and *γ*, *β*_1_ and *β*_2_ are the constants having values −0.1423, 1.0523, and 0.3334 respectively.

On the other hand, *V*_abs_ is the imaginary part. It takes into account all the inelastic processes and shows how much of the incident flux is lost through all the possible inelastic channels when the positron is scattered by the target. In the present calculation, electronic excitations, positronium (Ps) formation, and direct ionization are the inelastic channels considered. For the absorption potential we have adopted the parametric form proposed by Reid and Wadehra.^44^ It is given as follows:4
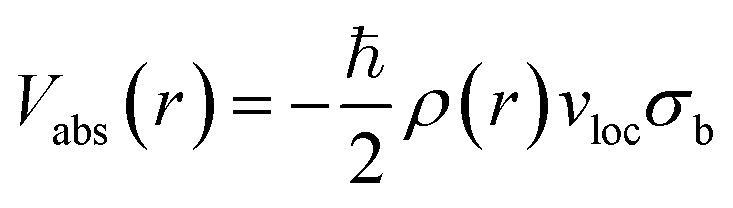
Here, *v*_loc_ denotes the local speed of the positron, and *σ*_b_ represents the binary collision cross section. The local speed can be determined from the incident energy, while the expression for the binary collision cross section was derived by Reid and Wadehra^44^ and is given as:5
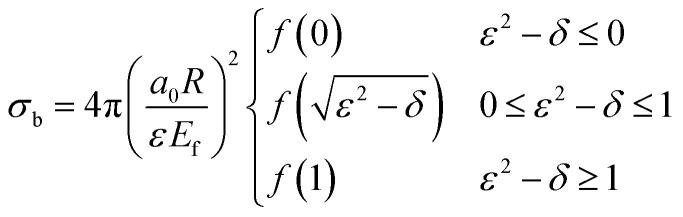
Here, *a*_0_ and *R* denotes the Bohr radius and the Rydberg constant, respectively. The function *f*(*x*) is defined as:6
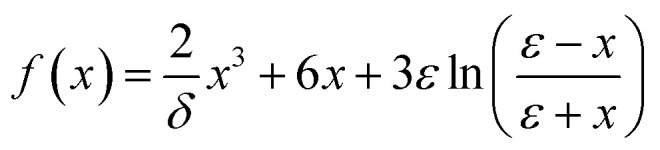


The parameters used in this expression are defined as 
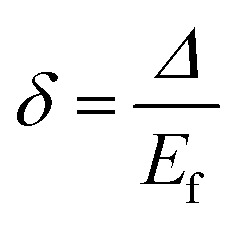
 and 
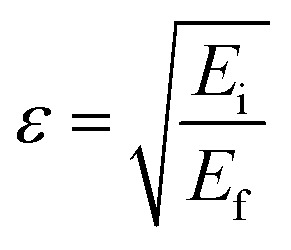
. Here, *Δ* represents the inelastic threshold, *E*_i_ is the incident energy of the projectile, and *E*_f_ is the Fermi energy associated with the target charge density.

The main challenge in the case of positron scattering is defining the inelastic threshold, below which all inelastic processes are prohibited. Reid and Wadehra proposed using the energy (*Δ*_p_) needed for Ps formation as the absorption threshold (*Δ*). However, this approach tends to slightly overestimate the total cross-section at higher energies. To address this problem, we modified Chiari's^45^ exponential form of the threshold by substituting the target's electronic excitation energy with its ionization potential (IP). We made this change because the positronium formation threshold for many targets nearly matches their electronic excitation energy, leading to inaccurate results. This work uses a modified form of the inelastic threshold given by,7
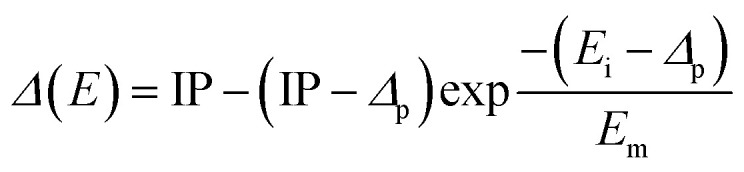


In the above equation, *Δ*_p_ is the energy level at which positronium (Ps) starts to form, and *E*_m_ is the energy level at which the absorption potential (*V*_abs_) generates the maximum cross section in the absence of Ps formation.

Unlike the electron scattering case, the presence of the Ps formation channel prevents the CSP-ic method from estimating the ionization cross section directly from the inelastic cross section. To overcome this, another inelastic cross section (*Q*_in_) is introduced, excluding the Ps formation channel. This new inelastic cross section is employed to calculate the direct ionization cross section (*Q*_iond_) using the well-known CSP-ic method. The Ps formation cross section (*Q*_ps_) is calculated using the equation,

8 *Q*_ps_(*E*_i_) = *Q*_inel_(*E*_i_) − *Q*_in_(*E*_i_)

and the total ionization cross section (*Q*_iont_) is determined by adding the *Q*_ps_ and *Q*_iond_, which is expressed as,

9 *Q*_iont_(*E*_i_) = *Q*_iond_(*E*_i_) + *Q*_ps_(*E*_i_)

The target properties such as ionization potential (IP), positronium formation threshold (*Δ*_p_) and polarizability (*α*) that are used in this work are presented in Table 1. All the target properties of the targets HCP, CP, PN and PO are taken from the CCCBDB database.^46^ The ionization potential values of these targets provided in the database are experimentally measured. However, since experimentally determined polarizability values are unavailable for all these targets, calculated polarizabilities through Density Functional Theory (wB97X-D) with the aug-cc-pVTZ basis set were used. The target properties of CCP radical are not available in the CCCBDB database. Therefore, the molecular geometry of the CCP was built using Avogadro molecular modelling software^48^ and optimized using Density Functional Theory (B3LYP)^49^ method along with basis set def2-QZVPP^50^ and auxiliary basis set def2/J.^51^ Geometry optimization along with ionization potential and polarizability calculations were performed using ORCA 5.0.1 (ref. 47) and visualized in Gabedit.^52^

**Table 1:** Target properties of all the phosphorus-bearing compounds studied in the present work. IP: ionization potential; *Δ*_p_: positronium formation threshold; *α*: polarizability

Target	IP (eV)	*Δ*_p_ (eV)	*α* (Å^3^)
HCP^46^	10.79	3.99	5.335
CCP^47^	9.19	2.39	7.175
CP^46^	10.50	3.70	5.576
PO^46^	8.39	1.59	4.002
PN^46^	11.88	5.08	4.158

## 3 Results

This section presents graphical representations of different cross-sections for positron impact scattering on the selected targets.

### 3.1 Total cross section

**Fig. 2:**
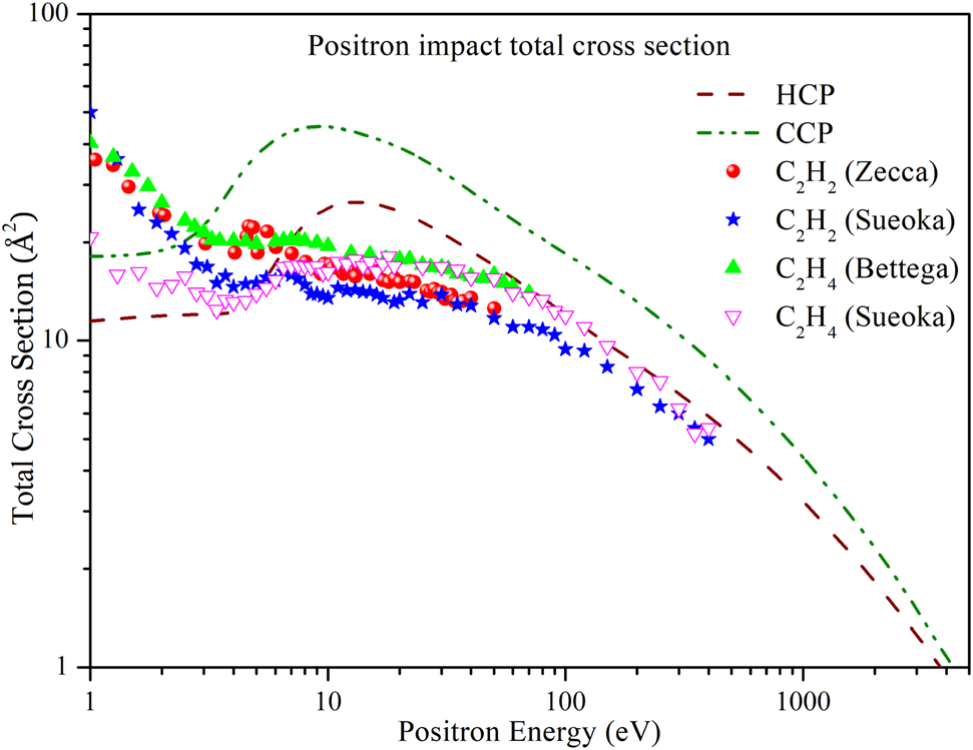
Positron impact total cross section of HCP and CCP compared with experimental *Q*_tot_ of C_2_H_2_ and C_2_H_4_. Dashed line: HCP; dashed dot dotted line: CCP; solid spheres: C_2_H_2_ measured by Zecca *et al.*;^53^ solid stars: C_2_H_2_ measured by Sueoka *et al.*;^54^ solid triangles: C_2_H_4_ measured by Bettega *et al.*;^55^ hollow inverted triangles: C_2_H_4_ measured by Sueoka *et al.*^56^

Fig. 2 shows the total cross section of HCP and CCP across a wide energy range from 1 eV to 5000 eV. The total cross section trend is similar for both targets, but CCP has a higher magnitude across the entire energy range due to its larger size. For HCP, the total cross section *Q*_tot_ initially shows a plateau at lower energies until the energy reaches the Ps formation threshold. Beyond this threshold, the cross section increases, peaking at 26.612 Å^2^ at 13 eV, and then decreases monotonically. Similarly, for CCP, *Q*_tot_ increases beyond the Ps formation threshold, reaching a maximum value of 45.242 Å^2^ at 9 eV, before decreasing monotonically at higher energies.

In the absence of previous experimental or theoretical studies on these targets, we compared the present results with those of acetylene (C_2_H_2_) and ethylene (C_2_H_4_) molecules. Acetylene is a linear molecule with a carbon–carbon triple bond, similar to the carbon-phosphorus triple bond in HCP. As a result, we used acetylene to compare HCP's total cross-section. We selected ethylene for comparison with CCP because it has a linear structure and a carbon–carbon double bond similar to CCP's.

Fig. 2 includes experimental total cross-section data for acetylene measured by Zecca *et al.*^53^ and Sueoka *et al.*^54^ Except at energies below 10 eV, there is qualitative agreement between the cross-section values of acetylene and HCP. In this study, rotational and vibrational cross sections were not considered, which may cause lower cross section values at energies below the Ps formation threshold. Furthermore, the study fails to adjust the experimental values for forward angle scattering, which could result in an overestimation of the cross-section values. In the intermediate energy range from 10 eV to 100 eV, the cross section of HCP is higher than that of acetylene, as expected due to its larger size. As energy increases, the difference in the magnitude of their cross-section decreases. The *Q*_tot_ measured by Sueoka *et al.*^54^ aligns well with the present *Q*_tot_ of HCP above 100 eV.

The figure also includes experimental total cross-section data for ethylene measured by Bettega *et al.*^55^ and Sueoka *et al.*^56^ The data reported by Bettega are much higher than the present results at lower energies. In our approach, rotational and vibrational contributions were not included, which may cause an underestimation of the total cross section (TCS) compared to experimental values. Sueoka's data align well with the present results at lower energies below the Ps formation threshold. At higher energies, the larger size of the phosphorus atom in CCP justifies a significantly higher cross section than that of ethylene.

**Fig. 3:**
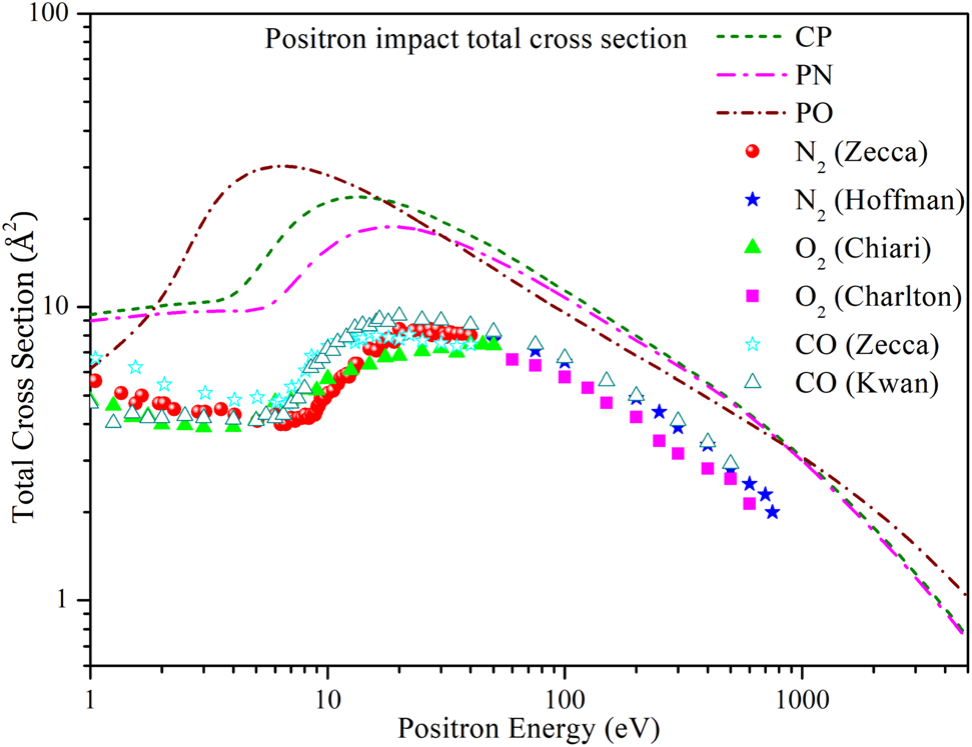
Positron impact total cross section of CP, PN, and PO compared with experimental *Q*_tot_ of N_2_, O_2_ and CO. Short dashed line: CP; dashed dotted line: PN; short dashed dotted line: PO; solid spheres: N_2_ measured by Zecca *et al.*;^53^ solid stars: N_2_ measured by Hoffman *et al.*;^57^ solid triangles: O_2_ measured by Chiari *et al.*;^45^ solid squares: O_2_ measured by Charlton *et al.*;^58^ hollow stars: CO measured by Zecca *et al.*;^53^ hollow triangles: CO measured by Kwan *et al.*^59^

Fig. 3 shows the energy dependence of the total cross section of CP, PN, and PO over a range of 1 eV to 5000 eV. The cross-sections of CP and PN show similar trends. For CP, the total cross section *Q*_tot_ initially exhibits a plateau at lower energies until it reaches the Ps formation threshold. Beyond this threshold, the cross section increases, peaking at 23.642 Å^2^ at 12 eV, and then decreases monotonically. Similarly, for PN, *Q*_tot_ increases beyond the Ps formation threshold, reaching a maximum value of 18.787 Å^2^ at 18 eV, before decreasing monotonically at higher energies. The cross section of PO increases with energy, peaking at 30.363 Å^2^ at 6 eV, and then decreases monotonically at higher energies. The graph shows that for all three targets, the *Q*_tot_ converges at higher energies.

In the absence of previous experimental or theoretical studies on these targets, we have chosen CO, N_2_, and O_2_ molecules for comparison. The CO molecule has a carbon–oxygen triple bond, similar to the phosphorus–carbon triple bond in CP. We compare the N_2_ molecule, which has a nitrogen–nitrogen triple bond, with PN, which has a similar phosphorus–nitrogen triple bond. Lastly, we compare the O_2_ molecule, which has an oxygen–oxygen double bond, to PO, which also has a similar phosphorus–oxygen double bond.

Fig. 3 includes experimental *Q*_tot_ data for CO measured by Zecca *et al.*^53^ and Kwan *et al.*,^59^ for N_2_ measured by Zecca *et al.*^53^ and Hoffman *et al.*,^57^ and for O_2_ measured by Chiari *et al.*^45^ and Charlton *et al.*^58^ The experimental cross sections show a similar trend to the present results, but the present *Q*_tot_ values have a higher magnitude across the entire energy range. A significant contribution to such differences comes from the positronium formation cross sections.

### 3.2 Positronium formation cross section

**Fig. 4:**
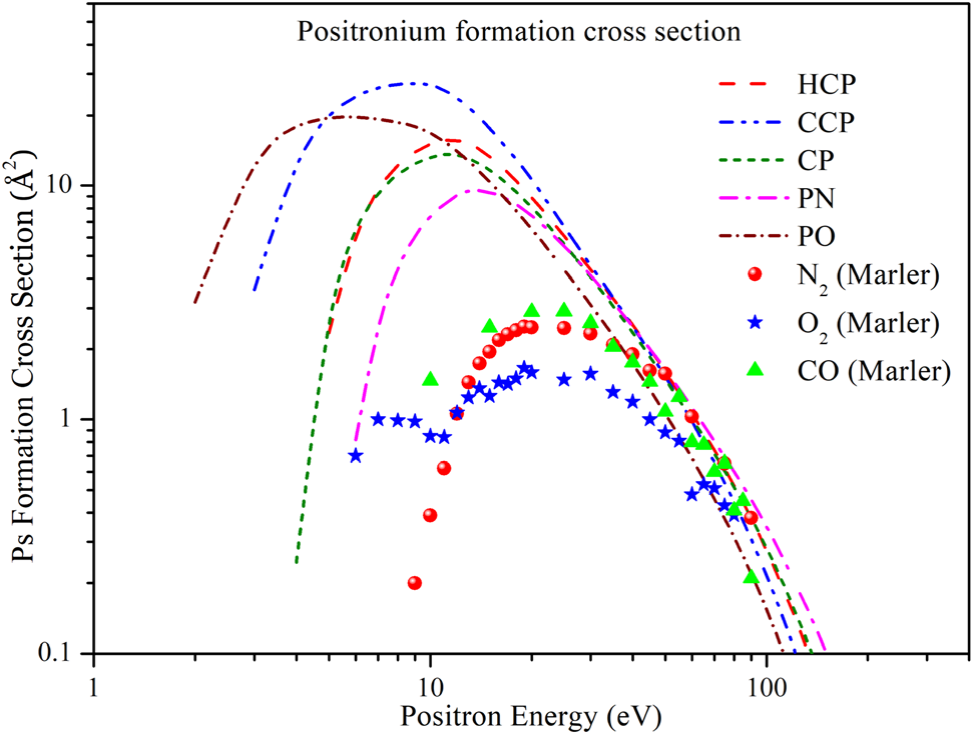
Positron impact Ps formation cross section of all phosphorus-bearing compounds compared with experimental *Q*_ps_ of N_2_, O_2_ and CO. Dashed line: HCP; dashed dot dotted line: CCP; short dashed line: CP; dashed dotted line: PN; short dashed dotted line: PO; solid spheres: N_2_;^60^ solid stars: O_2_;^60^ solid triangles: CO.^60^

In Fig. 4, the positronium formation cross section (*Q*_ps_) is plotted as a function of energy for all five targets. For all targets, the general trend of *Q*_ps_ is similar: it starts increasing above the Ps formation threshold, reaches a peak, and then decreases monotonically, fading out beyond 150 eV. According to the figure, *Q*_ps_ for HCP and CP almost overlap up to 7 eV. Beyond 7 eV, *Q*_ps_ for HCP is slightly higher than for CP, but they converge again beyond 30 eV. *Q*_ps_ for HCP reaches its maximum value of 15.704 Å^2^ at 12 eV, while for CP it peaks at 13.663 Å^2^ at 11 eV. For CCP and PO, *Q*_ps_ increases with energy, plateaus around 10 eV, and then decreases monotonically. The maximum *Q*_ps_ for CCP is 27.336 Å^2^ at 9 eV, and for PO it is 19.713 Å^2^ at 6 eV. For PN, *Q*_ps_ is relatively lower than for the other targets. It increases with energy, peaks at 9.576 Å^2^ at 14 eV, and then merges with the others beyond 30 eV.

Since there are no previous theoretical or experimental studies on these targets, we have included experimental *Q*_ps_ data for CO, N_2_, and O_2_ reported by Marler *et al.*^60^ The figure shows that the experimental *Q*_ps_ values are significantly lower than our results, which can be attributed to the presence of the phosphorus atom in the chosen targets.

### 3.3 Direct ionization cross section

**Fig. 5:**
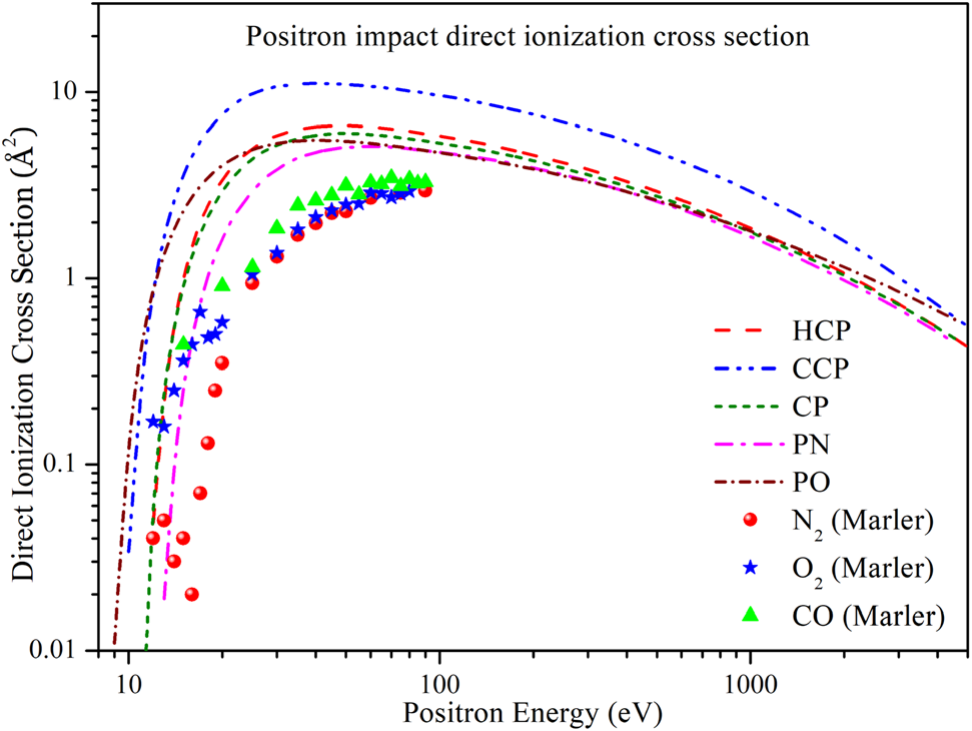
Positron impact direct ionization cross section of all phosphorus-bearing compounds compared with experimental *Q*_iond_ of N_2_, O_2_ and CO. Dashed line: HCP; dashed dot dotted line: CCP; short dashed line: CP; dashed dotted line: PN; short dashed dotted line: PO; solid spheres: N_2_;^60^ solid stars: O_2_;^60^ solid triangles: CO.^60^

In Fig. 5, the direct ionization cross section (*Q*_iond_) is plotted against energy. The general trend for *Q*_iond_ is similar for all targets: it increases rapidly above the ionization threshold, reaches a maximum value, and then decreases monotonically. CCP has a higher magnitude of cross section over the entire energy range, with *Q*_iond_ reaching its peak value of 11.127 Å^2^ at 40 eV. HCP and CP have almost overlapping cross sections across the entire energy range, although HCP has a slightly higher maximum value of *Q*_iond_ of 6.612 Å^2^ at 48 eV, compared to 6.015 Å^2^ at 50 eV for CP. At lower energies, PN shows a relatively lower magnitude of *Q*_iond_, reaching its maximum value of 5.128 Å^2^ at 60 eV. However, as energy increases above 100 eV, the differences in *Q*_iond_ diminish, and at higher energies, the values for HCP, CP, PO, and PN align with each other.

Because there were no previous theoretical or experimental studies on the selected targets, we included experimental direct ionization cross-section data for CO, N_2_, and O_2_ reported by Marler *et al.*^60^ However, these data are only available up to 100 eV. The figure shows that the trend of the experimental data is similar to our results, although the experimental *Q*_iond_ values are relatively lower. This discrepancy is likely due to the presence of heavier phosphorus atoms in the chosen targets.

### 3.4 Elastic cross section

**Fig. 6:**
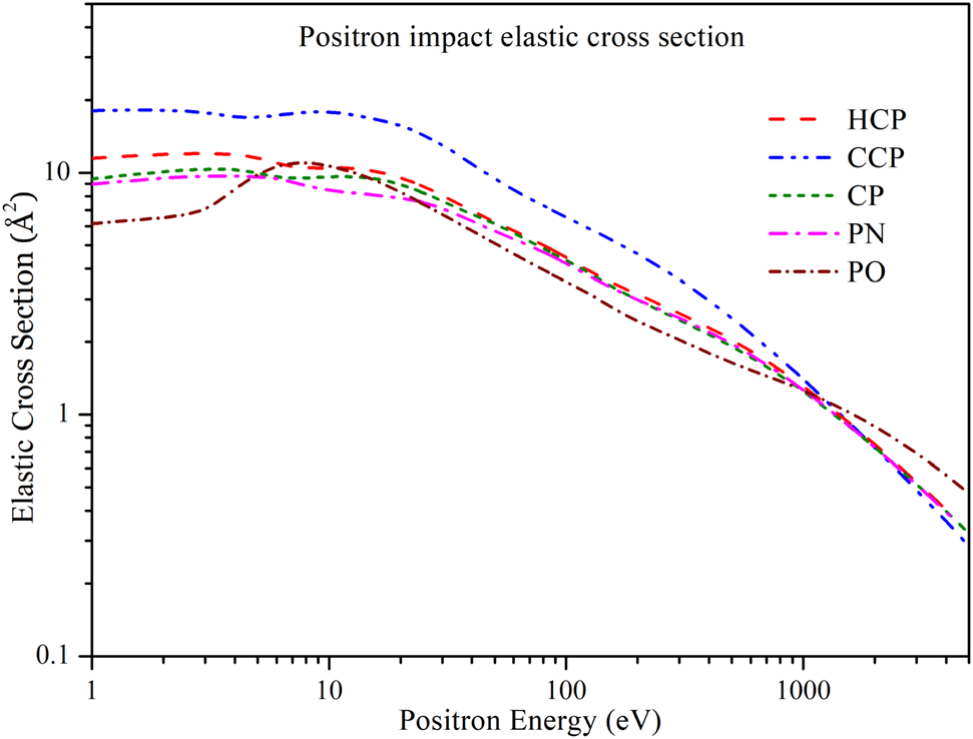
Positron impact elastic cross section of all phosphorus-bearing compounds. Dashed line: HCP; dashed dot dotted line: CCP; short dashed line: CP; dashed dotted line: PN; short dashed dotted line: PO.

In Fig. 6, the positron impact elastic cross section (*Q*_el_) is plotted as a function of energy over a range from 1 eV to 5000 eV. Due to the lack of available data for comparison in the literature, we compared the *Q*_el_ values of the targets with each other. For all targets, the general trend of *Q*_el_ is similar. CCP has a higher magnitude of *Q*_el_ up to 1000 eV, after which *Q*_el_ for CCP, HCP, CP, and PN align with each other. The PO's *Q*_el_ is slightly higher than the others above 1000 eV. For PO, *Q*_el_ exhibits a plateau at lower energies up to 3 eV, then increases to a peak value of 11.01 Å^2^ at 8 eV, and subsequently decreases to a minimum of 0.472 Å^2^ at 5000 eV. The other four targets (CCP, HCP, CP, and PN) show a plateau at lower energies up to 10 eV, beyond which *Q*_el_ starts decreasing. At lower energies, *Q*_el_ for HCP, CP, and PN have slightly different values, but these differences diminish as energy increases. Above 100 eV, *Q*_el_ for HCP, CP, and PN overlap with each other.

### 3.5 Total ionization cross section

**Fig. 7:**
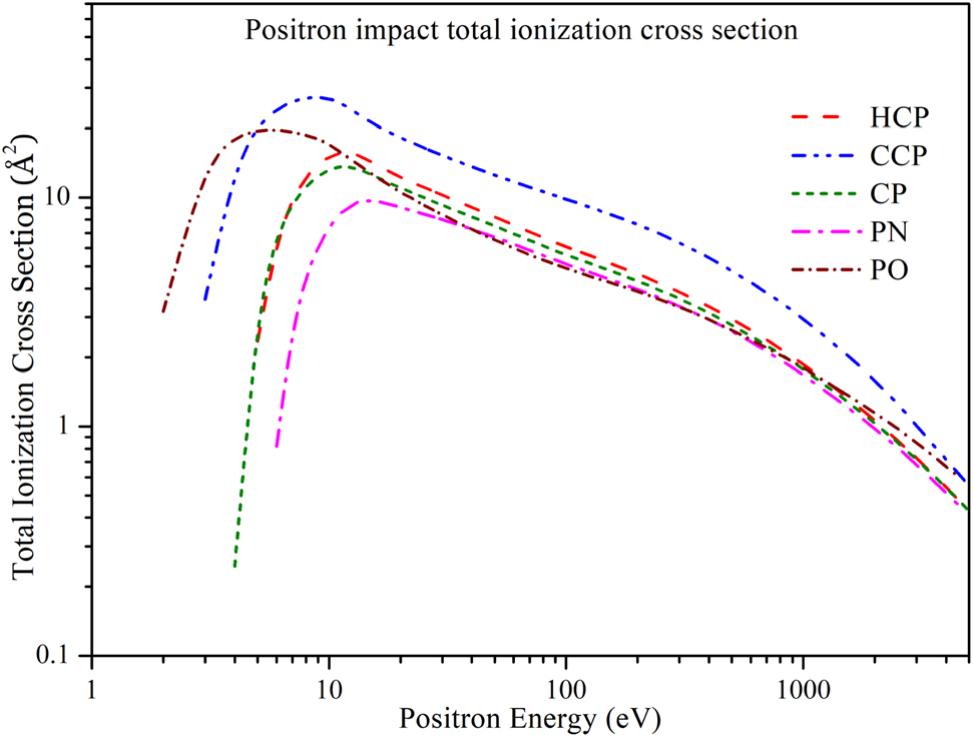
Positron impact total ionization cross section of all phosphorus-bearing compounds. Dashed line: HCP; dashed dot dotted line: CCP; short dashed line: CP; dashed dotted line: PN; short dashed dotted line: PO.

In Fig. 7, we can see how the positron impact total ionization cross section (*Q*_iont_) changes with energy from 1 eV to 5000 eV. Due to the absence of comparable data in the literature, we have compared the *Q*_iont_ values for the targets with each other. The general trend of *Q*_iont_ is similar across all targets: the cross sections increase above the Ps formation threshold, reach a maximum value, and then decrease as the energy increases. CCP exhibits a higher magnitude of *Q*_iont_ over the entire energy range, with a peak value of 27.336 Å^2^ at 9 eV. HCP and CP have overlapping *Q*_iont_ values at lower energies. However, HCP has a slightly higher maximum value of *Q*_iont_ than CP. *Q*_iont_ of HCP achieves its maximum value of 15.753 Å^2^ at 12 eV, whereas *Q*_iont_ for CP reaches its peak value of 13.666 Å^2^ at 11 eV. As the energy increases, the difference between their cross sections decreases. PN shows a relatively lower *Q*_iont_ at lower energies, reaching a maximum value of 9.679 Å^2^ at 14 eV. It then decreases and aligns with the *Q*_iont_ of PO from 40 eV to 400 eV. Beyond 400 eV, the *Q*_iont_ of PN aligns with that of HCP and CP. For PO, *Q*_iont_ reaches its maximum value at a lower energy compared to the other targets, peaking at 19.713 Å^2^ at 6 eV, and then decreases as the energy increases.

### 3.6 Differential cross section

**Fig. 8:**
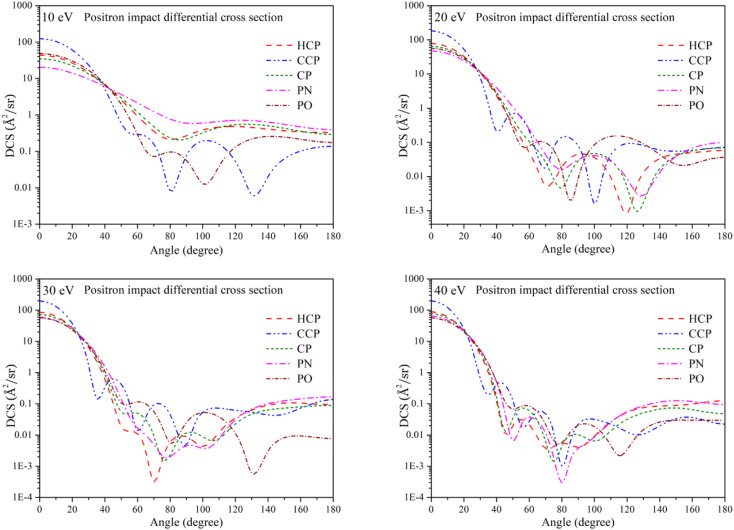
Positron impact differential cross sections of all phosphorus-bearing compounds at different impact energies. Dashed line: HCP; dashed dot dotted line: CCP; short dashed line: CP; dashed dotted line: PN; short dashed dotted line: PO.

Fig. 8 shows the elastic differential cross section (DCS) as a function of scattering angle at positron impact energies of 10, 20, 30, and 40 eV. The scattering angle is measured relative to the direction of the incident particle approaching the target molecule. Which means, the scattering angle is defined as the angle between the incoming positron's initial trajectory and the direction of the scattered positron after interacting with the target. The differential cross-section data explains the scattering of positrons at different angles during interactions with various compounds. The DCS data helps to model the scattering behavior of positrons, allowing for more accurate simulations of their paths. This modeling improves the precision of image reconstruction algorithms used in positron emission tomography (PET) imaging. At small scattering angles, the DCS curves initially had high values, indicating strong forward scattering. This occurs because of the coulombic interaction, *i.e.*, the long-range part of the potential. As the scattering angle increases, the probability of scattering typically decreases. The diminishing influence of long-range interactions at larger angles is responsible for this decrease. In addition to this decrease, an oscillatory behavior is observed for CCP and PO at 10 eV. As the energy increases, other targets start exhibiting this oscillation as well. This oscillatory nature of the DCS curves arises from the contributions of different partial waves, each associated with a specific angular momentum quantum number. When these partial waves scatter off a target, they interfere constructively or destructively depending on the scattering angle. At certain angles, partial waves interfere constructively, resulting in a peak in the differential cross section. At other angles, they interfere destructively, leading to dips. This interference pattern leads to the observed oscillations in the cross section. The point where the DCS curve reaches its minimum value represents the angle at which the scattering probability is the lowest. The increase in DCS toward 180° suggests a higher probability of backscattering. This can be attributed to polarization effects, in which the positron induces a dipole in the target, increasing scattering at larger angles.

## 4 Conclusion

This research investigates the positron impact cross sections for many phosphorus-containing compounds, such as HCP, CCP, CP, PN, and PO, at a wide energy range. The results provide important information about how positrons interact with these molecules. This helps us to understand their role in the interstellar medium and how they might be used in areas like positron emission tomography (PET) imaging. The total cross-section analysis (*Q*_tot_) showed that CCP always has the highest cross-section across all energy ranges. This is due to its larger molecular size compared to other targets. HCP and CP showed similar trends, with HCP having slightly higher cross-section values, while PN displayed relatively lower cross-sections. PO exhibited unique behavior, with an earlier peak and a more rapid decrease, indicating distinct interaction dynamics, possibly due to the presence of a double bond. Additionally, the higher cross section of PO may result from oxygen's higher atomic number compared to carbon and nitrogen. In the absence of previous data on these specific targets, we made comparisons with molecules such as acetylene, ethylene, CO, N_2_, and O_2_, which revealed qualitative agreements and highlighted the influence of the phosphorus atom in the observed increased cross-section values. This comparative approach allowed for a better understanding of the molecular similarities and differences in scattering behavior. For all targets, the positronium formation cross section (*Q*_ps_) and direct ionization cross section (*Q*_iond_) data showed similar trends, with clear peaks corresponding to different regions of energies. The presence of phosphorus in the targets significantly affected the *Q*_ps_ and *Q*_iond_, leading to higher values compared to the analogous molecules without phosphorus. These results were further supported by the elastic cross section (*Q*_el_) and total ionization cross section (*Q*_iont_) data, which showed consistent trends across the energy spectrum and among the different targets. Differential cross-section (DCS) analysis at various positron impact energies provided detailed information on the scattering behavior at different angles. At smaller angles, strong forward scattering was observed, reflecting the dominance of coulombic interactions. The oscillatory nature of the DCS curves, with peaks and dips resulting from constructive and destructive interference of partial waves, emphasizes the role of angular momentum in positron–molecule interactions. The variations in DCS across the different targets underscored the role of molecular structure in determining scattering behavior. In conclusion, this study not only fills a critical gap in the literature by providing cross-sectional data for previously unstudied phosphorus-bearing compounds but also underscores the importance of such data in understanding the physical and chemical processes in the interstellar medium. The results highlight the significance of the phosphorus atom in altering scattering dynamics, paving the way for further theoretical and experimental investigations. The present study also holds potential implications for practical applications, particularly in the refinement of imaging techniques in medical diagnostics.

## Data availability

See the ESI† for numerical cross-section data for the present targets (PDF).

## Conflicts of interest

There are no conflicts of interest.

## Supplementary Material

RA-014-D4RA06809B-s001
