# Supplementary material for: Positron scattering from interstellar phosphorus-bearing compounds
Source: RSC Adv. 2024 Dec 10;14(52):38855–63. doi: 10.1039/d4ra06809b (PMC11629107; doi:10.1039/d4ra06809b)
Supplement: RA-014-D4RA06809B-s001 [file RA-014-D4RA06809B-s001.pdf]

Supplementary Information (SI) for RSC Advances.
This journal is © The Royal Society of Chemistry 2024

Supplementary Information for

Positron scattering from interstellar phosphorus-bearing compounds

Irabati Chakraborty, Nafees Uddin and Bobby Antony

Email: bobby@iitism.ac.in

Table S1: Positron impact total cross section (Q_{tot}) of all the phosphorus-bearing compounds

Energy (eV)	Total cross section (Å^2)				
	HCP	CCP	CP	PN	PO
1	11.478	18.114	9.430	8.962	6.157
2	11.961	18.294	10.148	9.538	9.678
3	12.039	21.427	10.359	9.689	20.869
4	11.963	30.491	10.613	9.697	26.991
5	13.889	37.443	13.247	9.648	29.550
6	17.338	41.591	16.363	10.299	30.363
7	20.445	43.840	18.903	11.812	30.267
8	22.810	44.909	20.754	13.389	29.726
9	24.469	45.242	22.026	14.766	28.999
10	25.554	45.123	22.857	15.882	28.211
11	26.197	44.725	23.365	16.752	27.416
12	26.518	44.161	23.642	17.413	26.634
13	26.612	43.510	23.750	17.901	25.877
14	26.548	42.828	23.738	18.254	25.149
15	26.378	42.148	23.640	18.497	24.453
16	26.136	41.492	23.479	18.657	23.792
17	25.846	40.868	23.273	18.748	23.166
18	25.525	40.264	23.037	18.787	22.574
19	25.184	39.657	22.786	18.784	22.016
20	24.832	39.043	22.515	18.748	21.490
21	24.473	38.425	22.233	18.684	20.993
22	24.114	37.805	21.942	18.599	20.525
23	23.755	37.188	21.648	18.497	20.083
24	23.398	36.572	21.354	18.380	19.665
25	23.045	35.956	21.063	18.254	19.271
26	22.698	35.346	20.775	18.116	18.897
27	22.359	34.750	20.492	17.970	18.543
28	22.028	34.173	20.217	17.816	18.205
29	21.705	33.615	19.950	17.658	17.885
30	21.393	33.080	19.690	17.496	17.580
32	20.797	32.074	19.198	17.164	17.012
34	20.240	31.114	18.740	16.833	16.493
36	19.723	30.222	18.316	16.507	16.017
38	19.242	29.417	17.923	16.191	15.577
40	18.795	28.676	17.557	15.887	15.171
42	18.380	27.986	17.214	15.598	14.794

Energy (eV)	Total cross section (Å^2)				
	HCP	CCP	CP	PN	PO
44	17.993	27.338	16.889	15.323	14.444
46	17.633	26.728	16.580	15.063	14.116
48	17.294	26.153	16.282	14.817	13.808
50	16.975	25.618	15.992	14.585	13.521
55	16.238	24.441	15.304	14.054	12.873
60	15.571	23.439	14.677	13.574	12.311
65	14.968	22.566	14.109	13.125	11.817
70	14.424	21.794	13.597	12.704	11.382
75	13.933	21.107	13.134	12.314	10.994
80	13.487	20.487	12.715	11.955	10.646
85	13.084	19.922	12.333	11.623	10.330
90	12.712	19.401	11.985	11.318	10.044
95	12.366	18.921	11.665	11.035	9.782
100	12.044	18.477	11.372	10.767	9.542
110	10.850	17.676	10.850	10.274	9.112
120	10.384	16.963	10.384	9.832	8.742
130	9.959	16.324	9.959	9.446	8.411
140	9.576	15.749	9.576	9.110	8.109
150	9.239	15.228	9.239	8.811	7.839
160	8.937	14.751	8.937	8.542	7.597
170	8.664	14.311	8.664	8.298	7.378
180	8.415	13.903	8.415	8.076	7.178
190	8.187	13.522	8.187	7.869	6.994
200	7.977	13.165	7.977	7.680	6.830
250	7.114	11.661	7.114	6.895	6.155
300	6.467	10.490	6.467	6.298	5.655
350	5.952	9.541	5.952	5.813	5.243
400	5.525	8.753	5.525	5.407	4.907
450	5.165	8.087	5.165	5.061	4.630
500	4.854	7.516	4.854	4.759	4.396
600	4.341	6.586	4.341	4.257	4.019
700	3.930	5.860	3.930	3.852	3.721
800	3.595	5.277	3.595	3.520	3.477
900	3.314	4.798	3.314	3.242	3.272
1000	3.073	4.397	3.073	3.003	3.092
1500	2.258	3.077	2.258	2.196	2.457
2000	1.780	2.334	1.780	1.726	2.050
2500	1.465	1.851	1.465	1.420	1.762
3000	1.239	1.500	1.239	1.201	1.542

Energy (eV)	Total cross section (Å^2)				
	HCP	CCP	CP	PN	PO
3500	1.071	1.259	1.071	1.038	1.371
4000	0.939	1.081	0.939	0.911	1.232
4500	0.836	0.944	0.836	0.811	1.119
5000	0.750	0.836	0.750	0.728	1.024

Table S2: Positron impact Ps formation cross section (Q_{ps}) of all the phosphorus-bearing compounds

Energy (eV)	Positronium formation cross section (Å^2)				
	HCP	CCP	CP	PN	PO
1	0	0	0	0	0
2	0	0	0	0	3.18
3	0	3.593	0	0	14.043
4	0	13.497	0.246	0	18.387
5	2.351	20.486	3.276	0	19.659
6	6.391	24.287	6.747	0.82	19.713
7	9.844	26.21	9.411	2.655	19.287
8	12.329	27.07	11.244	4.536	18.716
9	13.996	27.336	12.452	6.139	18.006
10	15.062	26.974	13.23	7.412	16.844
11	15.694	25.638	13.663	8.389	15.474
12	15.704	23.754	13.569	9.127	14.075
13	15.169	21.662	13.105	9.521	12.742
14	14.331	19.579	12.441	9.576	11.517
15	13.357	17.628	11.684	9.411	10.414
16	12.353	15.869	10.9	9.11	9.432
17	11.383	14.314	10.128	8.728	8.562
18	10.478	12.943	9.393	8.307	7.794
19	9.648	11.724	8.718	7.871	7.115
20	8.895	10.632	8.086	7.435	6.516
21	8.213	9.65	7.502	7.011	5.982
22	7.599	8.764	6.964	6.605	5.509
23	7.046	7.983	6.471	6.219	5.086
24	6.546	7.296	6.022	5.854	4.707
25	6.092	6.689	5.613	5.518	4.367
26	5.681	6.151	5.241	5.205	4.061
27	5.307	5.675	4.903	4.911	3.783
28	4.968	5.257	4.595	4.636	3.531

Energy (eV)	Positronium formation cross section (Å^2)				
	HCP	CCP	CP	PN	PO
29	4.659	4.89	4.315	4.379	3.302
30	4.379	4.564	4.059	4.139	3.093
32	3.886	4.011	3.609	3.707	2.727
34	3.471	3.54	3.227	3.334	2.416
36	3.116	3.136	2.899	3.013	2.153
38	2.81	2.797	2.615	2.734	1.925
40	2.543	2.507	2.363	2.491	1.729
42	2.309	2.255	2.14	2.279	1.559
44	2.101	2.036	1.944	2.091	1.41
46	1.915	1.844	1.773	1.924	1.278
48	1.748	1.675	1.621	1.773	1.162
50	1.601	1.525	1.487	1.637	1.059
55	1.294	1.219	1.213	1.349	0.847
60	1.063	0.983	1.007	1.125	0.685
65	0.882	0.8	0.845	0.948	0.559
70	0.738	0.656	0.713	0.808	0.459
75	0.622	0.541	0.606	0.695	0.38
80	0.526	0.447	0.518	0.602	0.315
85	0.447	0.372	0.443	0.523	0.263
90	0.38	0.309	0.381	0.456	0.22
95	0.323	0.258	0.328	0.399	0.184
100	0.276	0.216	0.284	0.35	0.155
110	0.202	0.153	0.212	0.269	0.109
120	0.149	0.107	0.159	0.209	0.078
130	0.111	0.077	0.12	0.163	0.056
140	0.083	0.054	0.091	0.129	0.04
150	0.062	0.039	0.069	0.102	0.029
160	0.047	0.028	0.053	0.08	0.021
170	0.036	0.02	0.041	0.064	0.015
180	0.025	0.015	0.031	0.051	0.011
190	0.021	0.01	0.024	0.04	0.005
200	0.016	0.007	0.018	0.033	0.006
250	0.004	0.001	0.005	0.011	0.002
300	0.001	0.001	0.002	0.004	0
350	0.001	0	0	0.002	0.001
400	0	0	0	0	0
450	0	0	0	0.001	0
500	0	0	0	0	0
600	0	0	0	0	0

Energy (eV)	Positronium formation cross section (Å^2)				
	HCP	CCP	CP	PN	PO
700	0	0	0	0	0
800	0	0	0	0	0
900	0	0	0	0	0
1000	0	0	0	0	0
1500	0	0	0	0	0
2000	0	0	0	0	0
2500	0	0	0	0	0
3000	0	0	0	0	0
3500	0	0	0	0	0
4000	0	0	0	0	0
4500	0	0	0	0	0
5000	0	0	0	0	0

Table S3: Positron impact direct ionization cross section (Q_{iond}) of all the phosphorus-bearing compounds

Energy (eV)	Direct ionization cross section (Å^2)				
	HCP	CCP	CP	PN	PO
1	0	0	0	0	0
2	0	0	0	0	0
3	0	0	0	0	0
4	0	0	0	0	0
5	0	0	0	0	0
6	0	0	0	0	0
7	0	0	0	0	0
8	0	0	0	0	0
9	0	0	0	0	0.011
10	0	0.034	0	0	0.149
11	0.000	0.308	0.003	0	0.458
12	0.049	0.890	0.072	0	0.884
13	0.237	1.704	0.260	0.019	1.364
14	0.569	2.644	0.554	0.103	1.852
15	1.004	3.620	0.921	0.261	2.320
16	1.496	4.565	1.327	0.482	2.752
17	2.003	5.445	1.745	0.746	3.141
18	2.497	6.244	2.156	1.035	3.486
19	2.963	6.963	2.558	1.335	3.789
20	3.392	7.606	2.937	1.635	4.053

Energy (eV)	Direct ionization cross section (Å^2)				
	HCP	CCP	CP	PN	PO
21	3.781	8.179	3.287	1.927	4.282
22	4.130	8.687	3.606	2.206	4.480
23	4.450	9.124	3.895	2.469	4.650
24	4.735	9.493	4.155	2.715	4.795
25	4.988	9.804	4.386	2.947	4.921
26	5.210	10.063	4.591	3.166	5.027
27	5.406	10.278	4.772	3.367	5.118
28	5.577	10.451	4.932	3.551	5.196
29	5.726	10.589	5.073	3.719	5.261
30	5.856	10.699	5.197	3.871	5.316
32	6.067	10.853	5.404	4.133	5.400
34	6.225	10.974	5.564	4.346	5.457
36	6.344	11.064	5.691	4.516	5.493
38	6.432	11.109	5.790	4.654	5.514
40	6.498	11.127	5.868	4.763	5.522
42	6.546	11.126	5.928	4.850	5.520
44	6.579	11.109	5.970	4.920	5.511
46	6.601	11.082	5.997	4.977	5.497
48	6.612	11.047	6.012	5.023	5.478
50	6.612	11.005	6.015	5.059	5.456
55	6.580	10.878	5.989	5.114	5.391
60	6.511	10.737	5.931	5.128	5.319
65	6.427	10.590	5.860	5.113	5.242
70	6.334	10.441	5.783	5.076	5.165
75	6.240	10.294	5.704	5.029	5.089
80	6.146	10.150	5.625	4.977	5.015
85	6.058	10.008	5.547	4.923	4.944
90	5.975	9.869	5.471	4.870	4.875
95	5.895	9.735	5.397	4.820	4.810
100	5.817	9.604	5.327	4.771	4.746
110	5.668	9.353	5.201	4.676	4.627
120	5.527	9.114	5.083	4.584	4.518
130	5.391	8.886	4.969	4.493	4.419
140	5.261	8.673	4.861	4.403	4.331
150	5.138	8.471	4.756	4.317	4.247
160	5.022	8.279	4.654	4.234	4.167
170	4.911	8.097	4.557	4.154	4.091
180	4.807	7.924	4.465	4.078	4.017
190	4.708	7.761	4.376	4.004	3.947

Energy (eV)	Direct ionization cross section (Å^2)				
	HCP	CCP	CP	PN	PO
200	4.613	7.604	4.292	3.933	3.878
250	4.200	6.915	3.918	3.618	3.571
300	3.858	6.346	3.609	3.352	3.319
350	3.573	5.867	3.349	3.123	3.108
400	3.330	5.454	3.125	2.926	2.925
450	3.121	5.097	2.933	2.752	2.767
500	2.939	4.784	2.765	2.600	2.627
600	2.634	4.258	2.484	2.342	2.394
700	2.387	3.835	2.257	2.131	2.204
800	2.185	3.487	2.070	1.956	2.047
900	2.014	3.194	1.912	1.809	1.914
1000	1.869	2.946	1.777	1.680	1.798
1500	1.367	2.096	1.314	1.241	1.397
2000	1.067	1.594	1.038	0.980	1.149
2500	0.869	1.259	0.853	0.808	0.978
3000	0.728	1.010	0.720	0.682	0.849
3500	0.624	0.841	0.619	0.587	0.750
4000	0.543	0.718	0.540	0.513	0.669
4500	0.479	0.624	0.479	0.455	0.605
5000	0.427	0.551	0.427	0.407	0.551

Table S4: Positron impact elastic cross section (Q_{el}) of all the phosphorus-bearing compounds

Energy (eV)	Elastic cross section (Å^2)				
	HCP	CCP	CP	PN	PO
1	11.478	18.114	9.430	8.962	6.157
2	11.961	18.294	10.148	9.538	6.498
3	12.039	17.834	10.359	9.689	6.826
4	11.963	16.994	10.367	9.697	8.604
5	11.538	16.957	9.971	9.648	9.891
6	10.947	17.304	9.616	9.479	10.650
7	10.601	17.630	9.492	9.157	10.980
8	10.481	17.839	9.510	8.853	11.010
9	10.472	17.905	9.573	8.626	10.876
10	10.491	17.850	9.626	8.469	10.670
11	10.493	17.712	9.651	8.362	10.436
12	10.464	17.521	9.646	8.284	10.191

Energy (eV)	Elastic cross section ($Å^2$)				
	HCP	CCP	CP	PN	PO
13	10.403	17.299	9.613	8.221	9.943
14	10.314	17.063	9.558	8.167	9.694
15	10.205	16.824	9.486	8.115	9.447
16	10.082	16.592	9.401	8.065	9.204
17	9.949	16.371	9.305	8.014	8.968
18	9.811	16.154	9.202	7.962	8.739
19	9.669	15.925	9.069	7.909	8.520
20	9.526	15.683	8.931	7.855	8.310
21	9.383	15.429	8.793	7.798	8.110
22	9.240	15.166	8.654	7.740	7.920
23	9.081	14.897	8.517	7.681	7.740
24	8.923	14.623	8.381	7.620	7.570
25	8.767	14.343	8.249	7.544	7.409
26	8.615	14.063	8.119	7.457	7.256
27	8.468	13.788	7.992	7.369	7.112
28	8.325	13.521	7.870	7.281	6.974
29	8.186	13.263	7.752	7.193	6.844
30	8.052	13.017	7.638	7.106	6.720
32	7.799	12.558	7.424	6.933	6.490
34	7.564	12.081	7.228	6.766	6.281
36	7.349	11.626	7.049	6.606	6.089
38	7.151	11.236	6.886	6.453	5.912
40	6.969	10.884	6.739	6.309	5.749
42	6.802	10.560	6.605	6.174	5.598
44	6.649	10.255	6.481	6.048	5.458
46	6.511	9.967	6.364	5.930	5.327
48	6.385	9.695	6.252	5.821	5.204
50	6.268	9.447	6.141	5.721	5.090
55	6.004	8.924	5.875	5.504	4.833
60	5.766	8.500	5.629	5.318	4.611
65	5.549	8.141	5.404	5.145	4.417
70	5.353	7.830	5.202	4.983	4.248
75	5.175	7.559	5.020	4.833	4.098
80	5.014	7.319	4.856	4.694	3.964
85	4.866	7.103	4.708	4.566	3.842
90	4.724	6.906	4.574	4.447	3.732
95	4.589	6.725	4.452	4.332	3.631
100	4.461	6.559	4.339	4.220	3.539
110	4.220	6.261	4.131	4.009	3.373

Energy (eV)	Elastic cross section (Å^2)				
	HCP	CCP	CP	PN	PO
120	4.012	5.999	3.940	3.814	3.229
130	3.846	5.764	3.759	3.650	3.094
140	3.706	5.554	3.595	3.516	2.961
150	3.584	5.364	3.459	3.401	2.844
160	3.477	5.191	3.341	3.301	2.741
170	3.382	5.032	3.237	3.211	2.650
180	3.296	4.883	3.144	3.131	2.569
190	3.218	4.744	3.061	3.057	2.499
200	3.145	4.613	2.986	2.990	2.437
250	2.850	4.056	2.683	2.715	2.204
300	2.629	3.619	2.463	2.510	2.044
350	2.446	3.264	2.291	2.341	1.902
400	2.291	2.970	2.146	2.197	1.793
450	2.156	2.722	2.022	2.071	1.707
500	2.036	2.510	1.913	1.959	1.637
600	1.833	2.170	1.728	1.767	1.528
700	1.666	1.908	1.576	1.608	1.443
800	1.526	1.701	1.449	1.475	1.372
900	1.407	1.534	1.341	1.362	1.311
1000	1.305	1.396	1.247	1.265	1.256
1500	0.956	0.960	0.923	0.929	1.043
2000	0.752	0.730	0.731	0.732	0.892
2500	0.620	0.587	0.605	0.604	0.779
3000	0.526	0.487	0.515	0.514	0.690
3500	0.457	0.416	0.449	0.447	0.619
4000	0.404	0.362	0.397	0.395	0.561
4500	0.361	0.319	0.356	0.354	0.513
5000	0.326	0.285	0.322	0.320	0.472

Table S5: Positron impact total ionization cross section (Q_{iont}) of all the phosphorus-bearing compounds

Energy (eV)	Total ionization cross section (Å^2)				
	HCP	CCP	CP	PN	PO
1	0	0	0	0	0
2	0	0	0	0	3.18
3	0	3.593	0	0	14.043
4	0	13.497	0.246	0	18.387

Energy (eV)	Total ionization cross section (Å^2)				
	HCP	CCP	CP	PN	PO
5	2.351	20.486	3.276	0	19.659
6	6.391	24.287	6.747	0.82	19.713
7	9.844	26.21	9.411	2.655	19.287
8	12.329	27.07	11.244	4.536	18.716
9	13.996	27.336	12.452	6.138	18.017
10	15.062	27.008	13.23	7.412	16.993
11	15.694	25.946	13.666	8.389	15.932
12	15.753	24.644	13.641	9.127	14.959
13	15.406	23.366	13.365	9.54	14.106
14	14.9	22.223	12.995	9.679	13.369
15	14.361	21.248	12.605	9.672	12.734
16	13.849	20.434	12.227	9.592	12.184
17	13.386	19.759	11.873	9.474	11.703
18	12.975	19.187	11.549	9.342	11.28
19	12.611	18.687	11.276	9.206	10.904
20	12.287	18.238	11.023	9.07	10.569
21	11.994	17.829	10.789	8.938	10.264
22	11.729	17.451	10.570	8.811	9.989
23	11.496	17.107	10.366	8.688	9.736
24	11.281	16.789	10.177	8.569	9.502
25	11.08	16.493	9.999	8.465	9.288
26	10.891	16.214	9.832	8.371	9.088
27	10.713	15.953	9.675	8.278	8.901
28	10.545	15.708	9.527	8.187	8.727
29	10.385	15.479	9.388	8.098	8.563
30	10.235	15.263	9.256	8.01	8.409
32	9.953	14.864	9.013	7.84	8.127
34	9.696	14.514	8.791	7.68	7.873
36	9.46	14.2	8.590	7.529	7.646
38	9.242	13.906	8.405	7.388	7.439
40	9.041	13.634	8.231	7.254	7.251
42	8.855	13.381	8.068	7.129	7.079
44	8.68	13.145	7.914	7.011	6.921
46	8.516	12.926	7.770	6.901	6.775
48	8.36	12.722	7.633	6.796	6.64
50	8.213	12.53	7.502	6.696	6.515
55	7.874	12.097	7.202	6.463	6.238
60	7.574	11.72	6.938	6.253	6.004
65	7.309	11.39	6.705	6.061	5.801

Energy (eV)	Total ionization cross section (Å^2)				
	HCP	CCP	CP	PN	PO
70	7.072	11.097	6.496	5.884	5.624
75	6.862	10.835	6.310	5.724	5.469
80	6.672	10.597	6.143	5.579	5.33
85	6.505	10.38	5.990	5.446	5.207
90	6.355	10.178	5.852	5.326	5.095
95	6.218	9.993	5.725	5.219	4.994
100	6.093	9.82	5.611	5.121	4.901
110	5.87	9.506	5.413	4.945	4.736
120	5.676	9.221	5.242	4.793	4.596
130	5.502	8.963	5.089	4.656	4.475
140	5.344	8.727	4.952	4.532	4.371
150	5.2	8.51	4.825	4.419	4.276
160	5.069	8.307	4.707	4.314	4.188
170	4.947	8.117	4.598	4.218	4.106
180	4.832	7.939	4.496	4.129	4.028
190	4.729	7.771	4.4	4.044	3.952
200	4.629	7.611	4.31	3.966	3.884
250	4.204	6.916	3.923	3.629	3.573
300	3.859	6.347	3.611	3.356	3.319
350	3.574	5.867	3.349	3.125	3.109
400	3.33	5.454	3.125	2.926	2.925
450	3.121	5.097	2.933	2.753	2.767
500	2.939	4.784	2.765	2.6	2.627
600	2.634	4.258	2.484	2.342	2.394
700	2.387	3.835	2.257	2.131	2.204
800	2.185	3.487	2.07	1.956	2.047
900	2.014	3.194	1.912	1.809	1.914
1000	1.869	2.946	1.777	1.68	1.798
1500	1.367	2.096	1.314	1.241	1.397
2000	1.067	1.594	1.038	0.98	1.149
2500	0.869	1.259	0.853	0.808	0.978
3000	0.728	1.01	0.72	0.682	0.849
3500	0.624	0.841	0.619	0.587	0.75
4000	0.543	0.718	0.54	0.513	0.669
4500	0.479	0.624	0.479	0.455	0.605
5000	0.427	0.551	0.427	0.407	0.551

Table S6: Positron impact differential cross section (DCS) of all the phosphorus-bearing compounds at impact energy 10 eV

Angle (degree)	Differential cross section (Å$^2/sr$)				
	HCP	CCP	CP	PN	PO
0	44.023	124.922	34.963	20.404	48.457
5	42.667	119.701	33.999	19.919	47.113
10	38.862	105.221	31.285	18.560	43.298
15	33.309	84.620	27.291	16.568	37.596
20	26.918	61.938	22.627	14.250	30.812
25	20.557	40.904	17.880	11.884	23.779
30	14.875	24.029	13.501	9.671	17.208
35	10.230	12.276	9.766	7.723	11.598
40	6.718	5.266	6.788	6.081	7.203
45	4.241	1.819	4.554	4.738	4.056
50	2.599	0.544	2.969	3.662	2.019
55	1.562	0.280	1.895	2.811	0.854
60	0.931	0.298	1.195	2.144	0.294
65	0.557	0.277	0.750	1.628	0.093
70	0.346	0.175	0.473	1.237	0.064
75	0.240	0.062	0.310	0.952	0.087
80	0.206	0.004	0.225	0.760	0.101
85	0.222	0.021	0.199	0.644	0.089
90	0.268	0.087	0.215	0.588	0.059
95	0.328	0.158	0.261	0.577	0.028
100	0.387	0.200	0.324	0.594	0.010
105	0.435	0.199	0.392	0.627	0.015
110	0.468	0.162	0.456	0.662	0.041
115	0.484	0.108	0.507	0.692	0.084
120	0.485	0.057	0.541	0.710	0.134
125	0.473	0.020	0.557	0.712	0.182
130	0.454	0.005	0.554	0.700	0.221
135	0.431	0.007	0.536	0.674	0.247
140	0.408	0.022	0.506	0.638	0.259
145	0.386	0.042	0.470	0.595	0.258
150	0.368	0.064	0.430	0.550	0.248
155	0.354	0.085	0.392	0.507	0.232
160	0.343	0.102	0.357	0.468	0.215
165	0.336	0.117	0.329	0.436	0.199
170	0.332	0.129	0.309	0.412	0.186
175	0.330	0.136	0.296	0.398	0.178

Energy (eV)	Differential cross section (Å$^2/sr$)				
	HCP	CCP	CP	PN	PO
180	0.329	0.138	0.292	0.393	0.175

Table S7: Positron impact differential cross section (DCS) of all the phosphorus-bearing compounds at impact energy 20 eV

Angle (degree)	Differential cross section (Å$^2/sr$)				
	HCP	CCP	CP	PN	PO
0	78.493	184.169	64.967	47.398	56.441
5	74.353	171.444	61.905	45.412	54.045
10	63.233	137.917	53.614	40.014	47.469
15	48.320	94.941	42.294	32.569	38.257
20	33.160	54.734	30.413	24.605	28.236
25	20.368	25.264	19.881	17.306	18.958
30	11.132	8.436	11.742	11.351	11.433
35	5.382	1.530	6.214	6.955	6.061
40	2.301	0.106	2.925	3.990	2.724
45	0.885	0.487	1.228	2.150	0.972
50	0.325	0.786	0.479	1.092	0.247
55	0.126	0.616	0.199	0.526	0.061
60	0.052	0.264	0.104	0.241	0.076
65	0.018	0.036	0.060	0.105	0.111
70	0.004	0.010	0.029	0.043	0.105
75	0.007	0.091	0.008	0.019	0.065
80	0.021	0.155	0.003	0.014	0.022
85	0.037	0.147	0.013	0.021	0.001
90	0.046	0.087	0.030	0.032	0.011
95	0.046	0.026	0.044	0.040	0.045
100	0.037	0.001	0.049	0.042	0.089
105	0.024	0.014	0.043	0.038	0.129
110	0.011	0.049	0.031	0.029	0.152
115	0.002	0.080	0.017	0.018	0.156
120	0.000	0.095	0.006	0.009	0.144
125	0.005	0.093	0.001	0.003	0.120
130	0.014	0.081	0.002	0.002	0.092
135	0.025	0.068	0.008	0.006	0.065
140	0.034	0.059	0.018	0.014	0.044
145	0.041	0.055	0.029	0.025	0.029
150	0.045	0.055	0.040	0.039	0.022

Angle (degree)	Differential cross section ($Å^2/sr$)				
	HCP	CCP	CP	PN	PO
155	0.048	0.056	0.050	0.054	0.020
160	0.050	0.058	0.057	0.068	0.023
165	0.052	0.061	0.063	0.081	0.027
170	0.055	0.066	0.066	0.091	0.032
175	0.058	0.069	0.068	0.097	0.035
180	0.059	0.071	0.069	0.099	0.037

Table S8: Positron impact differential cross section (DCS) of all the phosphorus-bearing compounds at impact energy 30 eV

Angle (degree)	Differential cross section ($Å^2/sr$)				
	HCP	CCP	CP	PN	PO
0	86.885	199.089	73.551	59.508	57.278
5	80.856	181.158	68.984	56.171	54.159
10	65.286	135.925	57.059	47.387	45.876
15	45.873	82.773	41.790	35.944	34.915
20	28.016	39.325	27.065	24.584	23.819
25	14.743	13.245	15.338	15.113	14.414
30	6.567	2.378	7.449	8.278	7.587
35	2.391	0.056	2.988	3.977	3.359
40	0.658	0.443	0.920	1.631	1.176
45	0.114	0.731	0.192	0.547	0.300
50	0.013	0.490	0.042	0.144	0.084
55	0.014	0.146	0.050	0.035	0.097
60	0.013	0.006	0.054	0.016	0.124
65	0.005	0.045	0.034	0.010	0.107
70	0.000	0.108	0.012	0.005	0.061
75	0.002	0.106	0.001	0.002	0.021
80	0.007	0.054	0.002	0.002	0.003
85	0.010	0.009	0.008	0.003	0.010
90	0.009	0.003	0.013	0.005	0.028
95	0.006	0.028	0.012	0.005	0.046
100	0.004	0.058	0.009	0.004	0.054
105	0.006	0.074	0.006	0.004	0.051
110	0.012	0.074	0.007	0.008	0.040
115	0.023	0.067	0.013	0.017	0.025
120	0.036	0.062	0.023	0.032	0.012
125	0.049	0.058	0.036	0.050	0.004

Angle (degree)	Differential cross section (Å^2/sr)				
	HCP	CCP	CP	PN	PO
130	0.064	0.055	0.047	0.069	0.000
135	0.078	0.049	0.057	0.087	0.001
140	0.091	0.043	0.064	0.102	0.003
145	0.102	0.042	0.069	0.115	0.006
150	0.107	0.046	0.073	0.126	0.008
155	0.108	0.056	0.076	0.135	0.009
160	0.106	0.072	0.080	0.144	0.009
165	0.102	0.093	0.084	0.153	0.009
170	0.098	0.115	0.087	0.161	0.008
175	0.095	0.132	0.089	0.166	0.008
180	0.095	0.139	0.090	0.168	0.008

Table S9: Positron impact differential cross section (DCS) of all the phosphorus-bearing compounds at impact energy 40 eV

Angle (degree)	Differential cross section (Å^2/sr)				
	HCP	CCP	CP	PN	PO
0	89.589	200.840	77.693	64.628	57.256
5	82.070	179.054	71.871	60.210	53.529
10	63.369	126.311	57.175	48.938	43.942
15	41.529	69.107	39.381	34.995	31.879
20	23.070	27.689	23.385	22.020	20.380
25	10.705	6.927	11.736	12.052	11.318
30	3.997	0.551	4.775	5.592	5.316
35	1.091	0.116	1.429	2.084	2.005
40	0.163	0.553	0.234	0.551	0.548
45	0.005	0.470	0.005	0.069	0.102
50	0.025	0.162	0.046	0.003	0.058
55	0.036	0.011	0.081	0.029	0.090
60	0.024	0.028	0.067	0.042	0.090
65	0.009	0.066	0.032	0.032	0.059
70	0.003	0.056	0.007	0.014	0.024
75	0.004	0.020	0.001	0.002	0.005
80	0.006	0.000	0.005	0.000	0.005
85	0.005	0.008	0.011	0.002	0.013
90	0.004	0.025	0.011	0.004	0.022
95	0.005	0.034	0.008	0.005	0.024
100	0.009	0.033	0.006	0.008	0.020

Angle (degree)	Differential cross section (Å$^2/sr$)				
	HCP	CCP	CP	PN	PO
105	0.017	0.028	0.046	0.015	0.012
110	0.027	0.023	0.081	0.026	0.005
115	0.040	0.017	0.067	0.041	0.002
120	0.054	0.012	0.032	0.056	0.003
125	0.067	0.010	0.053	0.072	0.009
130	0.077	0.010	0.053	0.087	0.015
135	0.083	0.014	0.061	0.102	0.022
140	0.085	0.020	0.069	0.115	0.026
145	0.087	0.027	0.073	0.124	0.029
150	0.088	0.034	0.074	0.127	0.030
155	0.092	0.038	0.071	0.125	0.030
160	0.098	0.037	0.065	0.119	0.030
165	0.107	0.033	0.059	0.111	0.030
170	0.116	0.027	0.053	0.103	0.030
175	0.123	0.024	0.049	0.098	0.030
180	0.126	0.022	0.048	0.096	0.030
